# Minimally Invasive Nephrectomy for the Management of Polycystic Kidney Disease: The Hilum-First Technique

**DOI:** 10.3390/jcm14217485

**Published:** 2025-10-22

**Authors:** Amir Shweiki, Harbi Khalayleh, Michael Rivin, Suha Shabaneh, Abed Khalaileh, Ashraf Imam

**Affiliations:** 1 Organ Transplantation Unit, Department of Surgery, Hadassah Hebrew University Medical Center, Jerusalem 91120, Israel; 2 Faculty of Medicine, Hebrew University of Jerusalem, Jerusalem 91120, Israel; 3 Department of Surgery, Kaplan Hebrew University Medical Center, Rehovot 76100, Israel

**Keywords:** polycystic kidney disease, minimally invasive nephrectomy, hilum-first technique, early vascular control, robot-assisted nephrectomy, laparoscopic nephrectomy

## Abstract

**Background:** Nephrectomy in patients with polycystic kidney disease (PKD) is typically arduous due to the considerable size of the kidneys. The laparoscopic method has arisen as a minimally invasive substitute for open surgery. Nonetheless, conventional laparoscopic methods may be inadequate for tackling the distinct anatomical complexities of a large polycystic kidney. This study presents a unique method, “the hilum first technique”, specifically designed for nephrectomy in patients with PKD, emphasizing its safety and efficacy in addressing this intricate condition. **Methods:** A retrospective analysis of patients with PKD who underwent minimally invasive nephrectomy using “the hilum first technique” at our hospital between 2020 and 2025. Data on operative time, blood loss, conversion rates, hospital stay, and outcomes were analyzed to evaluate this technique’s safety and efficacy. **Results:** Minimally invasive nephrectomy using the “hilum first technique” was successfully performed in 16 cases; the mean age of patients was 56.3 years. Two of which were robot-assisted, in which one of them, a bilateral nephrectomy was done, with no conversions to open surgery, even for huge kidneys. The mean operative time was 159.6 min, with an estimated blood loss of 50 mL. Postoperatively, the median duration of hospital stay was 4 days (range: 3–10 days), and 75% of patients experienced no complications. Three patients (18.7%) were readmitted within 30 days due to surgical site infection, subcutaneous hematoma, and pneumonia. Seven patients (43.7%) underwent kidney transplantation within a median duration of 132 days post-nephrectomy. **Conclusions**: This retrospective study, although limited by a small sample size, demonstrated significant promise as a novel strategy for tackling the challenges of huge polycystic kidneys. The findings suggest its feasibility and safety, although further validation is required.

## 1. Introduction

Autosomal Dominant Polycystic Kidney Disease (ADPKD) is the most common and serious hereditary nephropathy and the fourth leading cause of end-stage renal disease (ESRD) worldwide. It affects roughly 1 in every 500 to 1000 people worldwide, accounting for an estimated 12 million affected persons [1,2]. The disease progression is linked to a variety of complications that affect patients’ quality of life and cause chronic discomfort, including refractory hypertension, urinary tract infections, nephrolithiasis, and hematuria [3]. Nephrectomy is often required in half of these cases when conservative management techniques are insufficient to relieve the condition or as preparation for kidney transplantation [4]. In recent years, minimally invasive techniques, such as laparoscopic and robot-assisted nephrectomy, have become more popular alternatives than traditional open nephrectomy, offering reduced complications and quicker recovery [5,6]. However, the widespread use of standard laparoscopic procedures has been constrained by the technical difficulties presented by patients with polycystic kidney disease (PKD) who have massively enlarged and cystic kidneys, and therefore other approaches like hand-assisted nephrectomy were developed [7]. We developed a novel approach, “the hilum first technique”, suitable for these unique cases that overcomes many of the technical obstacles.

In this article, we describe our experience with this novel approach, “the hilum first technique”, in a series of 16 ADPKD patients, detailing the surgical steps, perioperative outcomes, and postoperative recovery. We also analyze the safety, feasibility, and efficacy of this approach, highlighting its potential to improve surgical management for patients with huge polycystic kidneys.

## 2. Materials and Methods

This study is a retrospective analysis of a case series involving a total of 16 consecutive patients with Autosomal Dominant Polycystic Kidney Disease (ADPKD) who underwent minimally invasive nephrectomy using a novel “the hilum first technique” at our hospital between November 2020 and February 2025, without application of any exclusion criteria. Preoperative evaluation included detailed imaging such as CT or MRI to assess kidney size, cyst distribution, and vascular anatomy. Surgical key steps were described in detail. Demographic, operative, and postoperative data were obtained and analyzed. The primary outcomes assessed the safety and feasibility of the novel technique, while secondary outcomes included perioperative morbidity and patient recovery. Descriptive statistics were calculated using basic spreadsheet tools. Continuous variables were reported as means, medians, and ranges, while categorical data were summarized as counts and percentages. Postoperative complications were classified using the Clavien–Dindo system. Due to the small sample size, no inferential statistical tests were performed. This study was approved by the local Institutional Review Board (IRB).

Artificial intelligence (AI) tools were used to assist with language editing and formatting of this manuscript. All scientific content, data analysis, and interpretation were conducted by the authors.

### 2.1. Surgical Technique

#### 2.1.1. Positioning

The patient was placed in a lateral decubitus position on the Contralateral side with a 30-degree table break to open the costophrenic angle and facilitate access to the kidney. The surgeon and the assistant stand anterior to the patient. Video monitors are located at the head of the operating table.

#### 2.1.2. Port Placement for Laparoscopy

Preoperative imaging, including CT and MRI, was utilized to assess kidney size, cyst distribution, and anatomical relationships, guiding port placement and dissection. In our study, 4 to 5 ports were placed and distributed as follows:

- A 12 mm camera port placed in the periumbilical region on the side of the planned nephrectomy.
- A 12 mm port placed under the costal arch and another two or three 5 mm ports placed in the mid and lower flank on the side of the nephrectomy.
- Ports were inserted under vision, and pneumoperitoneum was maintained using CO_2_ to 15 mmHg.

Note: Port Placement for Robot-Assisted Technique

Since we use a da Vinci xi, we used four 8 mm incisions in the same line, 1 to 2 cm next to the umbilicus, to the side of the nephrectomy.

#### 2.1.3. The Hilum-First Technique

**Figure 1:**
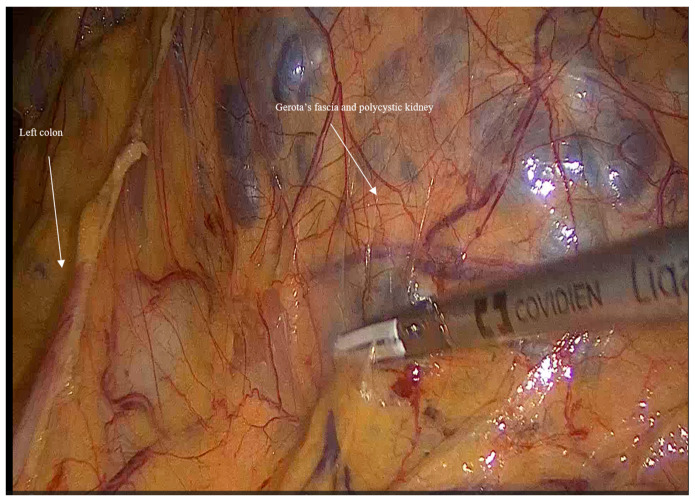
Shows left colon mobilization and exposing the left Gerota’s fascia and left polycystic kidney.

- Mobilization: Initially, the left colon was mobilized laterally, and Gerota’s fascia was identified. Careful dissection was performed to reveal the kidney Figure 1.

An immediate approach to the kidney hilum was achieved by dissecting the inferior border of the kidney at the level of the hilum, then more blunt dissection was performed posteriorly to separate the kidney from the psoas muscle.

**Figure 2:**
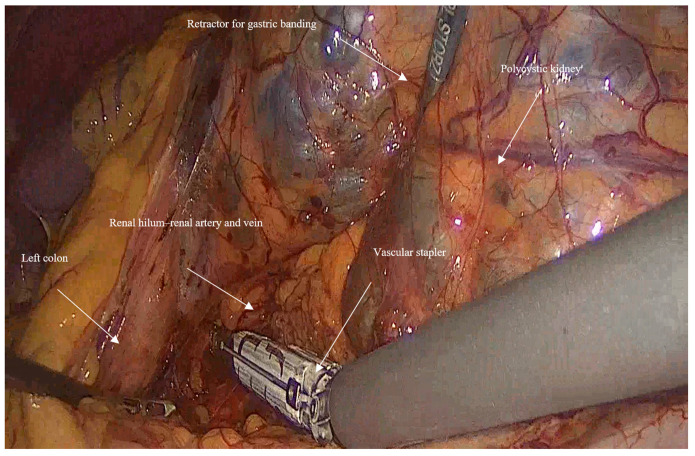
Shows the early control on vasculature by using the vascular stapler early on, following the mobilization of the left colon, retraction of the left kidney, and dissection of the inferior border, to ligate the renal artery and vein together at the kidney hilum.

- Vascular control and ligation: The renal artery and vein were clamped together close to the hilum of the kidney using a vascular stapler (Signia^TM^ with Tri-Staple^TM^ reinforced reloads, Medtronic, Minneapolis, MN, USA). Following that, a dissection of the rest of the inferior border of the kidney is completed, and then the ureter is ligated using Hem-o-lock™ clips (Weck, Research Triangle, NC, USA; Figure 2).

#### 2.1.4. Enucleation of the Kidney

The kidney was enucleated from the surrounding fatty tissue, ensuring minimal trauma to adjacent tissue and preserving the adrenal gland. Controlled aspiration rather than rupture of large cysts was done during dissection of the kidney from the spleen and the adrenal gland, if needed.

#### 2.1.5. Extraction Technique

The excised kidney was placed into a specimen bag and removed through a Pfannenstiel incision using a wound protector. If the patient had undergone a past renal transplant to the same side as the nephrectomy, the kidney transplant incision was used for kidney extraction. The large cysts were ruptured and suctioned inside the bag and outside of the abdomen during extraction through the incision to prevent field contamination and chemical peritonitis that may cause postoperative ileus.

#### 2.1.6. Closure

Sequential closure of the fascia and skin layers was performed with absorbable sutures. Drains were not routinely placed, but they were taken into consideration based on intraoperative findings.

### 2.2. Challenges and Tips

**Figure 3:**
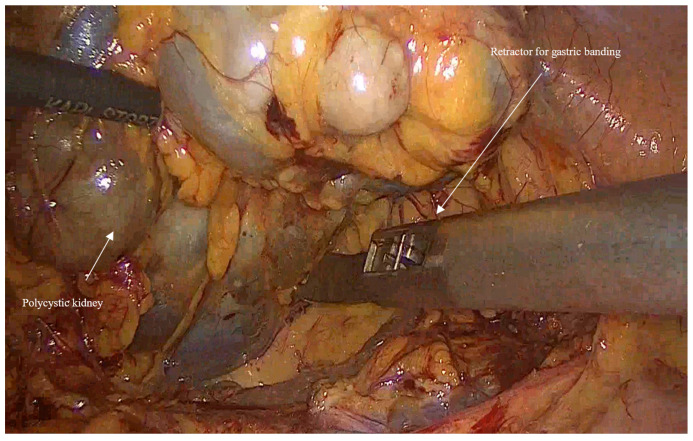
Shows the retraction of the kidney using a retractor for gastric banding to get early access to the kidney hilum.

1. In huge kidney with previous infections where controlled aspiration may be hazardous, a sufficient retraction with a retractor for Gastric Banding (Karl Storz 30,623 GB), which has a 0-to-90-degree angle of its tip and can provide an efficient retraction and elevation of the huge kidney from the psoas muscle and assist in gaining access to the hilum of the kidney (Figure 3).
2. When performing a right nephrectomy, the release of the lateral ligaments of the right liver is important and should be performed early in the surgery to facilitate a good exposure to the vena cava.
3. For bilateral nephrectomies, a robot-assisted procedure may be better in terms of fewer incisions since it can be done using 4 robotic incisions in the midline (two above and two below the umbilicus)

**Figure 4:**
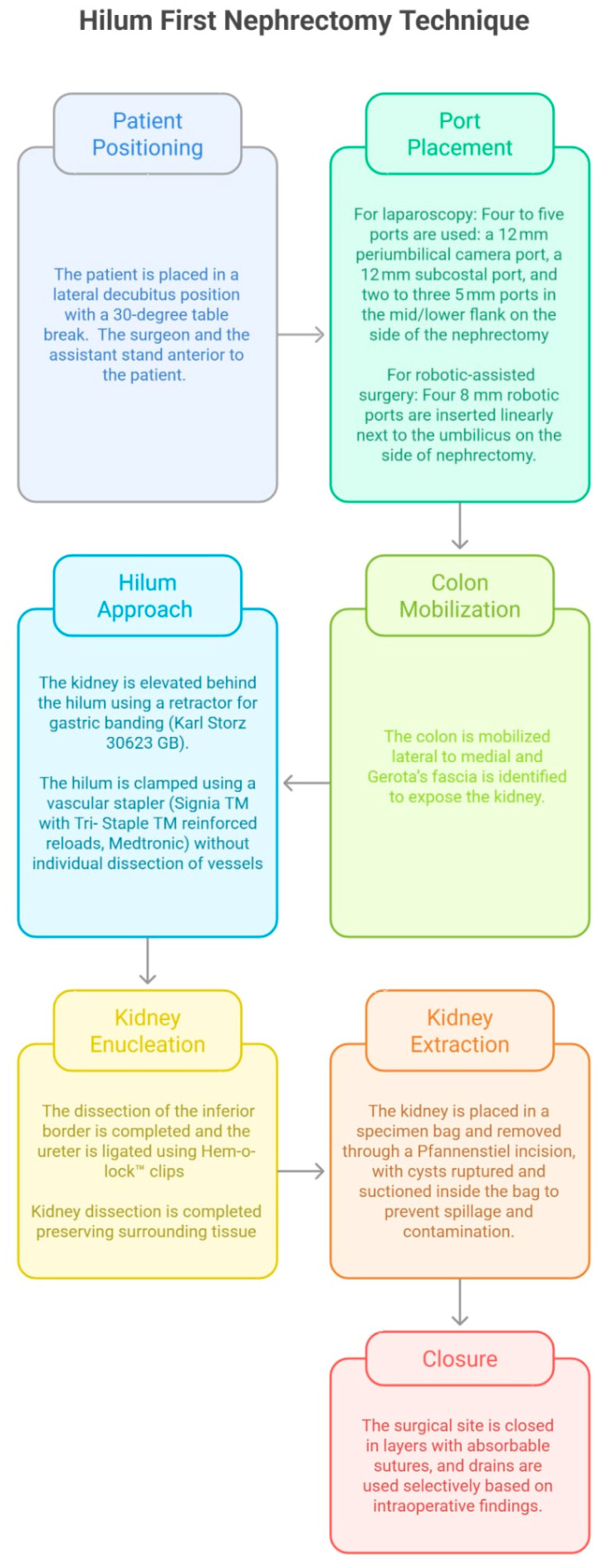
The key steps of the hilum-first technique.

A schematic diagram outlining the key steps of the hilum-first technique is illustrated in Figure 4.

## 3. Patient Information, Clinical Findings & Diagnostic Assessment

The study cohort included 16 cases of ADPKD who underwent minimally invasive nephrectomy, two of which were robot-assisted. The sample comprised 12 males and 4 females, with a mean age of 56.3 years (range: 42–74). Common comorbidities included hypertension (*n* = 12), diabetes mellitus (*n* = 3), dyslipidemia (*n* = 5), obstructive sleep apnea (*n* = 1), and ischemic heart disease (*n* = 1). Eight patients (50%) were on dialysis preoperatively, while the remaining eight (50%) had previously undergone kidney transplantation, reflecting the advanced stage of their disease.

Nephrectomies were performed on the left kidney in 12 cases, the right kidney in 3 cases, as one of them was robot-assisted, and bilaterally in 1 case (robot-assisted). Most of the patients had multiple symptoms before surgery. The most common was flank pain and abdominal bloating in 50% of cases, followed by recurrent urinary tract infections (37.5%), hemorrhagic cysts with hematuria (15%), and rectourethral fistula (6.25%). Seven patients (43.7%) underwent nephrectomy as a preparatory measure for anticipated kidney transplantation, and they all received transplants within a mean interval of 230 days (range: 1 month–1.5 years), (Table 1).

Preoperative imaging, including computed tomography (CT) and magnetic resonance imaging (MRI), was utilized to assess kidney size, cyst distribution, and anatomical relationships. Laboratory tests, including perioperative serum creatinine and hemoglobin levels, were evaluated.

## 4. Therapeutic Intervention & Outcome

As for the perioperative parameters (Table 2), the mean operative time for laparoscopic nephrectomy was 159.6 min (range: 102–266), with estimated minimal blood loss around 50 mL. Two patients required one unit of blood transfusion each due to low preoperative hemoglobin levels. None of the patients required conversion to open surgery, even for huge kidneys weighing up to 2170 g, according to pathology reports. According to the pathology report, the mean weight of the excised kidneys was 1177.2 g (range: 364–2170), with a mean volume of 2428.4 cm^3^ (range: 570–4320), taking into consideration that most of the cysts were ruptured and suctioned during the extraction of the kidney. Postoperatively, the average hospital stay was 5.4 ± 1.9 days, with a median duration of 4 days (range: 3–10 days). During the in-hospital postoperative period, twelve patients (75%) experienced no complications, while four (25%) developed postoperative issues, including thrombosis of the arteriovenous fistula (2 patients), ileus (1 patient), and hyperkalemia requiring dialysis (1 patient). Moreover, three patients (18.7%) were readmitted within 30 days due to surgical site infection, subcutaneous hematoma, or pneumonia. According to the Clavien–Dindo classification, and taking into consideration the readmitted patients, complications were graded as follows: Grade 1 (*n* = 2), Grade 2 (*n* = 2), Grade 3a (*n* = 2), and Grade 4a (*n* = 1) (Table 3). Pathological examination revealed papillary renal cell carcinoma (RCC) in one patient (6.2%). Patients who underwent transplantation after nephrectomy had a median waiting duration and follow-up of 132 days, as follow-up was discontinued after transplantation, whereas in the non-transplant group, the median follow-up was 590 days. No late complications were reported in the follow-up period of both groups. No mortality was reported until the time of writing this article.

## 5. Discussion

The surgical treatment of ADPKD has changed dramatically over time, moving from open nephrectomy to less invasive approaches. While open nephrectomy is still common in certain European centers for kidneys weighing more than 2000 g due to technical difficulties [4], laparoscopic techniques developed by Elashry et al. [8] and hand-assisted methods introduced by Schmidlin et al. [9] have proven feasibility across diverse settings [10]. A 2015 systematic review confirms this evolutionary shift, demonstrating superior outcomes for the laparoscopic approach even for large kidneys [11].

Unlike nephrectomy for normal-sized kidney or oncological indications, where anatomy is preserved and the procedure follows a standardized, reproducible approach [12], ADPKD nephrectomy is technically demanding. The massively enlarged kidney distorts normal landmarks, displaces adjacent organs, and restricts operative space, while adhesions from infections or cyst rupture further complicate dissection. These factors increase operative time, blood loss, and complication risk, sometimes necessitating hand-assisted approaches or conversion to open surgery [13,14,15].

Our study demonstrates the safety and efficiency of our novel approach, “the hilum first technique” in totally transperitoneal laparoscopic or robot-assisted nephrectomy in patients with APKD.

The hilum-first technique differs from conventional approaches in the sequence of dissection. In our method, the renal hilum is approached immediately after colonic mobilization to secure early vascular control, without prior cyst puncture or complete kidney mobilization. At the hilum, the renal artery and renal vein are divided together using a vascular stapler, after which the kidney is dissected from its attachments. In contrast, conventional approaches generally begin with controlled cyst decompression to improve operative exposure, followed by superior and inferior dissection, mobilization of the kidney from adjacent structures such as the spleen and adrenal gland, and subsequent stepwise individual dissection and clamping of the ureter, renal vein, and renal artery.

In our series, the absence of intraoperative complications or conversions to open surgery is in contrast with previously reported rates of 25.6% and 4.6%, respectively, for traditional transperitoneal laparoscopic nephrectomy [16]. Compared with other studies summarized in a recently published meta-analysis [10], our mean operative time of 159 min was shorter than most pure laparoscopic series (179–522 min) and also lower than reported HALN cohorts. Only a single study has reported a shorter operative duration; however, this involved substantially smaller kidneys (a mean of 403 g) compared with 1177.2 g in our series. Our estimated blood loss was minimal, and blood transfusion was limited to patients with pre-existing anemia rather than intraoperative bleeding, consistent with generally low transfusion rates across published series. Major complications (Clavien–Dindo grade ≥ III) occurred in 18.7% of patients, predominantly related to dialysis-dependent status, as we assume that the arteriovenous fistula thrombosis in 2 cases and the episode of hyperkalemia that needed emergent dialysis are more likely associated with the patients’ underlying dialysis status, rather than the nephrectomy itself, and this remains within the broad reported range of 4–35%.

Regarding hospitalization, although some early series reported shorter lengths of stay, our median hospitalization of 4 days (range 3–10) aligns with contemporary practice. These findings suggest that our technique achieved perioperative outcomes that are at least similar to, and in some aspects superior to, those reported studies.

A modest learning curve was observed in our series, as postoperative complications, including ileus, subcutaneous hematoma, and surgical site infection, occurred in cases performed during the first 2 years of this study. A clear operative time trend was difficult to assess, likely due to variability of kidney size, prior surgical history, and the use of robotic assistance in 2 later cases. Early in the learning curve, some unintentional cyst ruptures were encountered, which led to spillage. One patient who developed ileus and another readmitted with surgical site infection were among these cases, but the limited sample size precludes any definitive conclusions regarding an association. Importantly, no late complications were reported either in the group who later underwent transplant, or the non-transplant group. Post-transplant events were beyond the scope of this study.

Recent advancements in robotic technology further support the feasibility of minimally invasive approaches for complex urologic procedures. A recent systematic review of innovations in robot-assisted genitourinary surgery, highlighting single-port robotic platforms and three-dimensional virtual modeling for surgical planning and intraoperative guidance. These developments have been shown to enhance surgical precision, improve safety, reduce invasiveness, and accelerate patient recovery [17]. In the context of ADPKD, robot-assisted nephrectomy has similarly shown promise, especially for large kidneys due to its improved dexterity and visualization [18,19]. In our study, the choice between laparoscopic and robot-assisted nephrectomy was based on surgeon judgment, considering kidney size, anticipated procedural complexity, and the indication for bilateral nephrectomy. Robot-assisted procedures were performed in cases with smaller kidneys and less complex dissection, reflecting the recent introduction of the robotic platform at our center, as well as in bilateral nephrectomies, since it could be safely done using the same incisions while still applying “the hilum first technique”. The link between ADPKD and renal cell carcinoma (RCC) remains a topic of debate. A Taiwanese nationwide cohort study involving 8692 individuals and a 2024 meta-analysis confirmed an increased but low absolute risk of renal cell carcinoma in patients with ADPKD, with an estimated prevalence of ~5.7% per person (4.3% per kidney) [20,21]. In our series, one out of 16 cases (6.2%) revealed previously undiagnosed papillary renal cell carcinoma. This finding highlights the importance of cautious specimen handling and the possible risks associated with morcellation, which may impair histological evaluation and raise the risk of tumor cell spread [22].

In this series, the weights of the removed kidneys ranged from 364 to 2170 g according to pathology reports, taking into account cyst rupture during extraction. Although our maximum specimen weight was less than 2500 g, these findings are consistent with Collini et al.’s who conducted the largest series to date on laparoscopic nephrectomy for massive polycystic kidneys (>2500 g) [7], emphasizing that this approach, together with surgeon experience and planning, is feasible across a wide spectrum of renal sizes. The hilum-first technique is potentially applicable beyond high-volume centers, as early access and control of the vessels in the renal hilum facilitates safer and more efficient dissection. The approach is largely independent of arterial or venous anatomical variations, as it allows direct access to the hilum without the need for meticulous dissection or isolation of individual vessels, thereby simplifying adoption. Successful implementation, however, necessitates a thorough understanding of renal anatomy and advanced laparoscopic expertise. When performed by an experienced laparoscopic transplant or urologic surgeon, this approach can be safely applied in lower-volume centers. The main limitation of this technique is the presence of massively enlarged polycystic kidneys, which may restrict safe visualization of the renal hilum. In our series, this challenge was effectively addressed using a retractor for Gastric Banding (Karl Storz 30,623 GB), which allowed safe and effortless elevation of the kidney, ensuring secure access to the hilum even in very large kidneys.

Our findings support adopting “the hilum first technique” as a standard minimally invasive approach for ADPKD. Among the limitations of our study are its retrospective design and relatively small sample size. Nonetheless, our results align with current literature and offer valuable insights into the feasibility of minimally invasive nephrectomy in ADPKD.

## 6. Conclusions

Within the limitations of this small retrospective series, early vascular control at the kidney hilum may be beneficial and contribute to more favorable operative times and reduced risk of intraoperative complications. Although there is a substantial learning curve, this method can also be used for robotically assisted nephrectomy. Future multicenter prospective studies are needed to further validate these findings and establish evidence-based guidelines for surgical management in ADPKD.

## Figures and Tables

**Table 1:** Preoperative assessment.

Parameter	Value (*n* = 16)
Sex (male:female)	12:4
Mean age (years)	56.3
Preoperative dialysis (*n*)	8
Preoperative kidney transplantation	8
Operative side (left:right:bilateral)	12:3:1
Mean preoperative hemoglobin levels	12
Previous abdominal surgery	12
Comorbidities HTN ESRD DM Dyslipidemia Gout AFib IHD OSA	12 8 3 5 1 1 1 1
Preoperative presentations Abdominal and flank pain Preparation for transplant Recurrent UTIs Hemorrhagic renal cyst, hematuria Recto-urethral fistula Shortness of breath	8 7 6 4 1 1

HTN: Hypertension, ESRD: End Stage Renal Disease, UTI: Urinary Tract Infection, DM: Diabetes Mellitus, AFib: Atrial Fibrillation, IHD: Ischemic Heart Disease, OSA: Obstructive Sleep Apnea.

**Table 2:** Intraoperative and postoperative assessment.

Parameter	Value (*n* = 16)
Mean duration of procedure (minutes)	159.6
Estimated blood loss	Minimal (up to 50 mL)
Blood transfusion (units) 0 1	14 2
Mean kidney weight according to pathology reports (grams)	1177.2 (364–2170)
Mean kidney volume according to pathology reports (cm^3^)	2428.4 (570–4320)
Histopathology Malignancy (papillary RCC, T1NxMx)	1
Median duration of postoperative hospital stays (days)	4 (3–10)
Mean postoperative hemoglobin levels (g/dL)	11.2
Postoperative complications during the in-hospital postoperative period No complications Thrombosis of AV fistula requiring radiological intervention Ileus Hyperkalemia requiring dialysis	12 2 1 1
30-day readmission SSI Subcutaneous hematoma Pneumonia	3 1 1 1
Median time from nephrectomy to transplant (days)	132

RCC: Renal Cell Carcinoma, AV: Arteriovenous, SSI: Surgical Site Infection.

**Table 3:** Patients’ complications according to Clavien–Dindo classification.

Clavien–Dindo Grade	*n*
Grade I	2
Grade II	2
Grade IIIa	2
Grade IIIb	0
Grade IVa	1
Grade IVb	0
Grade V	0

## Data Availability

The original contributions presented in this study are included in the article. Further inquiries can be directed to the corresponding author.
